# 2-Amino-5-methyl­pyridinium 2-hy­droxy­benzoate

**DOI:** 10.1107/S1600536810030928

**Published:** 2010-08-11

**Authors:** Ching Kheng Quah, Madhukar Hemamalini, Hoong-Kun Fun

**Affiliations:** a X-ray Crystallography Unit, School of Physics, Universiti Sains Malaysia, 11800 USM, Penang, Malaysia

## Abstract

In the title compound, C_6_H_9_N_2_^+^·C_7_H_5_O_3_^−^, the protonated 2-amino-5-methyl­pyridinium cation and the 2-hy­droxy­benzoate anion are both essentially planar, with maximum deviations of 0.026 (2) and 0.034 (1) Å, respectively. The anion is stabilized by an intra­molecular O—H⋯O hydrogen bond, which forms an *S*(6) ring motif. In the solid state, the anions are linked to the cations *via* pairs of inter­molecular N—H⋯O hydrogen bonds forming *R*_2_^2^(8) ring motifs. The crystal structure is further stabilized by N—H⋯O and C—H⋯O inter­actions which link the mol­ecules into chains along [010]. A π–π stacking inter­action [centroid–centroid-distance = 3.740 (2) Å] is also observed.

## Related literature

For background to and the applications of carb­oxy­lic acids, see: Miller & Orgel (1974 ▶); Kvenvolden *et al.* (1971 ▶); Desiraju (1989 ▶); MacDonald & Whitesides (1994 ▶). For applications of salicylic acid, see: Singh & Vijayan (1974 ▶); Patel *et al.* (1988 ▶). For related structures, see: Quah *et al.* (2008 ▶; 2010*a* ▶,*b* ▶). For bond-length data, see: Allen *et al.* (1987 ▶). For hydrogen-bond motifs, see: Bernstein *et al.* (1995 ▶).
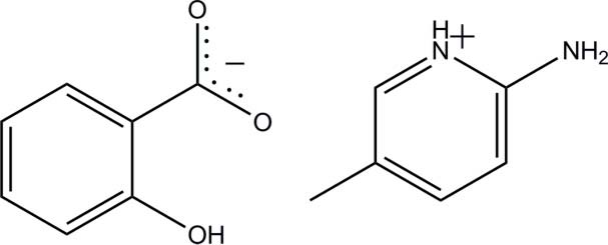


## Experimental

### 

#### Crystal data

C_6_H_9_N_2_^+^·C_7_H_5_O_3_^−^
*M* *_r_* = 246.26
Monoclinic, 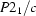

*a* = 13.211 (7) Å
*b* = 7.170 (4) Å
*c* = 14.324 (7) Å
β = 104.668 (11)°
*V* = 1312.6 (12) Å^3^
*Z* = 4
Mo *K*α radiation
μ = 0.09 mm^−1^
*T* = 297 K
0.42 × 0.19 × 0.10 mm

#### Data collection

Bruker SMART APEXII DUO CCD area-detector diffractometer
Absorption correction: multi-scan (*SADABS*; Bruker, 2009 ▶) *T*_min_ = 0.963, *T*_max_ = 0.991
14312 measured reflections
3797 independent reflections
2233 reflections with *I* > 2σ(*I*)
*R*_int_ = 0.028

#### Refinement

*R*[*F* ^2^ > 2σ(*F* ^2^)] = 0.043
*wR*(*F* ^2^) = 0.134
*S* = 1.01
3797 reflections
219 parameters
All H-atom parameters refined
Δρ_max_ = 0.14 e Å^−3^
Δρ_min_ = −0.15 e Å^−3^

### 

Data collection: *APEX2* (Bruker, 2009 ▶); cell refinement: *SAINT* (Bruker, 2009 ▶); data reduction: *SAINT*; program(s) used to solve structure: *SHELXTL* (Sheldrick, 2008 ▶); program(s) used to refine structure: *SHELXTL*; molecular graphics: *SHELXTL*; software used to prepare material for publication: *SHELXTL* and *PLATON* (Spek, 2009 ▶).

## Supplementary Material

Crystal structure: contains datablocks global, I. DOI: 10.1107/S1600536810030928/bt5314sup1.cif

Structure factors: contains datablocks I. DOI: 10.1107/S1600536810030928/bt5314Isup2.hkl

Additional supplementary materials:  crystallographic information; 3D view; checkCIF report

## Figures and Tables

**Table 1:** Hydrogen-bond geometry (Å, °)

*D*—H⋯*A*	*D*—H	H⋯*A*	*D*⋯*A*	*D*—H⋯*A*
N1—H1*N*1⋯O3	1.03 (2)	1.65 (2)	2.678 (2)	174.6 (13)
N2—H1*N*2⋯O2^i^	0.884 (18)	1.987 (17)	2.852 (2)	165.4 (14)
N2—H2*N*2⋯O2	0.97 (2)	1.90 (2)	2.872 (2)	179 (2)
O1—H1*O*1⋯O3	1.03 (2)	1.55 (2)	2.515 (2)	155 (2)
C5—H5*A*⋯O1^ii^	0.961 (14)	2.598 (14)	3.518 (3)	160.2 (10)

Symmetry codes: (i) 
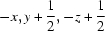
; (ii) 
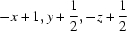
.

**Fig. 1.**
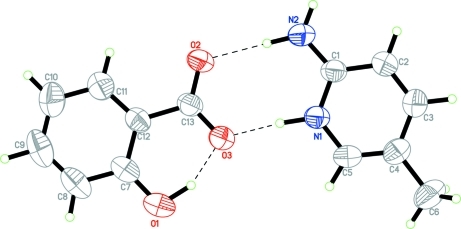
The molecular structure of the title compound showing 50% probability displacement ellipsoids for non-H atoms and the atom-numbering scheme. Intramolecular interactions are shown in dashed lines.

**Fig. 2.**
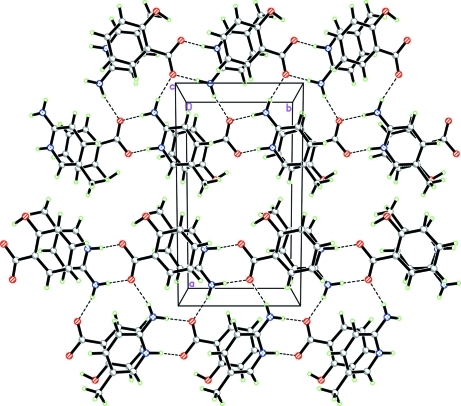
The crystal structure of the title compound viewed along the c axis.

## supplementary crystallographic information

### Comment

Hydrogen bonding has been established as the most effective tool for constructing sophisticated assemblies because of its strength and directionality. Carboxylic acids are believed to have existed in the prebiotic earth (Miller & Orgel, 1974; Kvenvolden *et al.*, 1971) and they exhibit characteristic intermolecular interactions and aggregation patterns. Also carboxyl groups have been used as primary building blocks in the design of crystal structures (Desiraju, 1989; MacDonald & Whitesides, 1994). Salicylic acid, a well known analgesic, and its complexes with a few drug molecules such as antipyrine (Singh & Vijayan, 1974) and sulfadimidine (Patel *et al.*, 1988) were already reported in the literature. The present study is aimed at investigating the supramolecular interactions of the title compound, (I).

The asymmetric unit of title compound (Fig. 1), contains a protonated 2-amino-5-methylpyridinium cation and a 2-hydroxybenzoate anion. In the 2-amino-5-methylpyridinium cation, a wide angle [122.26 (13)°] is subtended at the protonated N1 atom. The 2-amino-5-methylpyridinium cation and 2-hydroxybenzoate anion are essentially planar, with a maximum deviation of 0.026 (2) Å for atom C6 and 0.034 (1) Å for atom O3, respectively. The diheral angle between these two planes is 4.78 (5)°, indicating they are nearly parallel to each other. The anion is stabilized by an intramolecular O1–H1O1···O3 hydrogen bond, which forms an S(6) ring motif (Bernstein *et al.*, 1995).

In the solid state (Fig. 2), the anions are linked to the cations *via* intermolecular N1–H1N1···O3 and N2–H2N2..O2 hydrogen bonds forming *R*_2_^2^(8) ring motifs. The crystal structure is further stabilized by N2–H1N2···O2 and C5–H5A···O1 interactions. The molecules are linked by these interactions into chains along [010]. π–π stacking interactions with short intermolecular distance [3.740 (2) Å] between symmetry-related N1/C1—C5 (centroid *Cg*1) and C7—C12 (centroid *Cg*2) [symmetry code: *x*, 1 + *y*, *z*] are also observed.

### Experimental

A hot methanol solution (20 ml) of 2-amino-5-methylpyridine (54 mg, Aldrich) and salicylic acid (34.5 mg, Merck) was mixed and warmed over a magnetic stirrer hotplate for a few minutes. The resulting solution was allowed to cool slowly at room temperature and crystals of the title compound appeared after a few days.

### Refinement

All H atoms were located in a difference Fourier map and refined freely.

### Crystal data

**Table:**

C_6_H_9_N_2_^+^·C_7_H_5_O_3_^−^	*F*(000) = 520
*M**_r_* = 246.26	*D*_x_ = 1.246 Mg m^−^^3^
Monoclinic, *P*2_1_/*c*	Mo *K*α radiation, λ = 0.71073 Å
Hall symbol: -P 2ybc	Cell parameters from 3026 reflections
*a* = 13.211 (7) Å	θ = 3.2–26.8°
*b* = 7.170 (4) Å	µ = 0.09 mm^−^^1^
*c* = 14.324 (7) Å	*T* = 297 K
β = 104.668 (11)°	Block, yellow
*V* = 1312.6 (12) Å^3^	0.42 × 0.19 × 0.10 mm
*Z* = 4	

### Data collection

**Table:**

Bruker SMART APEXII DUO CCD area-detector diffractometer	3797 independent reflections
Radiation source: fine-focus sealed tube	2233 reflections with *I* > 2σ(*I*)
graphite	*R*_int_ = 0.028
φ and ω scans	θ_max_ = 30.0°, θ_min_ = 2.9°
Absorption correction: multi-scan (*SADABS*; Bruker, 2009)	*h* = −18→18
*T*_min_ = 0.963, *T*_max_ = 0.991	*k* = −10→10
14312 measured reflections	*l* = −20→19

### Refinement

**Table:**

Refinement on *F*^2^	Primary atom site location: structure-invariant direct methods
Least-squares matrix: full	Secondary atom site location: difference Fourier map
*R*[*F*^2^ > 2σ(*F*^2^)] = 0.043	Hydrogen site location: inferred from neighbouring sites
*wR*(*F*^2^) = 0.134	All H-atom parameters refined
*S* = 1.01	*w* = 1/[σ^2^(*F*_o_^2^) + (0.0607*P*)^2^ + 0.0959*P*] where *P* = (*F*_o_^2^ + 2*F*_c_^2^)/3
3797 reflections	(Δ/σ)_max_ < 0.001
219 parameters	Δρ_max_ = 0.14 e Å^−^^3^
0 restraints	Δρ_min_ = −0.15 e Å^−^^3^

### Special details

**Table:**

Geometry. All e.s.d.'s (except the e.s.d. in the dihedral angle between two l.s. planes) are estimated using the full covariance matrix. The cell e.s.d.'s are taken into account individually in the estimation of e.s.d.'s in distances, angles and torsion angles; correlations between e.s.d.'s in cell parameters are only used when they are defined by crystal symmetry. An approximate (isotropic) treatment of cell e.s.d.'s is used for estimating e.s.d.'s involving l.s. planes.
Refinement. Refinement of *F*^2^ against ALL reflections. The weighted *R*-factor *wR* and goodness of fit *S* are based on *F*^2^, conventional *R*-factors *R* are based on *F*, with *F* set to zero for negative *F*^2^. The threshold expression of *F*^2^ > σ(*F*^2^) is used only for calculating *R*-factors(gt) *etc*. and is not relevant to the choice of reflections for refinement. *R*-factors based on *F*^2^ are statistically about twice as large as those based on *F*, and *R*- factors based on ALL data will be even larger.

### Fractional atomic coordinates and isotropic or equivalent isotropic displacement parameters (Å^2^)

**Table:**

	*x*	*y*	*z*	*U*_iso_*/*U*_eq_	
N1	0.25056 (7)	0.79968 (17)	0.30963 (8)	0.0491 (3)	
N2	0.07534 (9)	0.7373 (2)	0.28908 (10)	0.0664 (4)	
C1	0.15649 (8)	0.84904 (19)	0.32288 (9)	0.0485 (3)	
C2	0.15072 (10)	1.0168 (2)	0.37198 (9)	0.0542 (3)	
C3	0.23752 (10)	1.1242 (2)	0.40264 (10)	0.0561 (3)	
C4	0.33511 (10)	1.0709 (2)	0.38749 (9)	0.0544 (3)	
C5	0.33759 (9)	0.9075 (2)	0.34103 (9)	0.0523 (3)	
C6	0.43050 (14)	1.1912 (3)	0.41915 (16)	0.0791 (5)	
O1	0.40974 (7)	0.27586 (19)	0.17167 (9)	0.0806 (4)	
O2	0.10956 (7)	0.41138 (14)	0.18462 (8)	0.0703 (3)	
O3	0.27788 (7)	0.48394 (15)	0.21915 (9)	0.0710 (3)	
C7	0.32912 (10)	0.1574 (2)	0.13606 (10)	0.0581 (4)	
C8	0.35051 (15)	−0.0109 (3)	0.09641 (12)	0.0760 (5)	
C9	0.27204 (17)	−0.1348 (3)	0.05976 (13)	0.0830 (5)	
C10	0.17009 (17)	−0.0959 (3)	0.06012 (13)	0.0799 (5)	
C11	0.14807 (12)	0.0712 (2)	0.09921 (11)	0.0635 (4)	
C12	0.22624 (9)	0.19952 (19)	0.13827 (9)	0.0494 (3)	
C13	0.20156 (9)	0.37630 (19)	0.18311 (10)	0.0529 (3)	
H2A	0.0850 (11)	1.050 (2)	0.3827 (10)	0.067 (4)*	
H3A	0.2348 (12)	1.246 (3)	0.4373 (11)	0.074 (5)*	
H5A	0.3970 (10)	0.854 (2)	0.3237 (9)	0.053 (3)*	
H6A	0.4887 (16)	1.146 (3)	0.3935 (14)	0.107 (7)*	
H6B	0.4150 (17)	1.318 (4)	0.3879 (17)	0.131 (9)*	
H6C	0.4491 (16)	1.208 (3)	0.4862 (19)	0.123 (8)*	
H8A	0.4216 (15)	−0.033 (3)	0.1009 (13)	0.097 (6)*	
H9A	0.2895 (14)	−0.256 (3)	0.0343 (13)	0.098 (6)*	
H10A	0.1156 (15)	−0.181 (3)	0.0375 (14)	0.102 (6)*	
H11A	0.0773 (12)	0.101 (2)	0.1003 (10)	0.068 (4)*	
H1N1	0.2564 (11)	0.678 (3)	0.2733 (11)	0.073 (4)*	
H1N2	0.0141 (13)	0.771 (2)	0.2980 (12)	0.078 (5)*	
H2N2	0.0862 (13)	0.626 (3)	0.2540 (13)	0.087 (5)*	
H1O1	0.3730 (16)	0.382 (3)	0.1984 (15)	0.116 (7)*	

### Atomic displacement parameters (Å^2^)

**Table:**

	*U*^11^	*U*^22^	*U*^33^	*U*^12^	*U*^13^	*U*^23^
N1	0.0397 (5)	0.0494 (7)	0.0589 (6)	0.0009 (5)	0.0137 (4)	−0.0009 (5)
N2	0.0401 (5)	0.0620 (9)	0.0988 (9)	−0.0039 (5)	0.0209 (6)	−0.0157 (7)
C1	0.0396 (5)	0.0504 (8)	0.0560 (7)	0.0017 (5)	0.0131 (5)	0.0018 (6)
C2	0.0480 (6)	0.0565 (9)	0.0592 (7)	0.0039 (6)	0.0158 (6)	−0.0028 (6)
C3	0.0614 (7)	0.0537 (9)	0.0520 (7)	−0.0011 (7)	0.0121 (6)	−0.0041 (7)
C4	0.0524 (7)	0.0579 (9)	0.0508 (7)	−0.0090 (6)	0.0092 (5)	0.0044 (6)
C5	0.0401 (6)	0.0607 (9)	0.0574 (7)	−0.0017 (6)	0.0143 (5)	0.0045 (7)
C6	0.0653 (9)	0.0833 (14)	0.0848 (12)	−0.0264 (10)	0.0118 (9)	−0.0071 (11)
O1	0.0495 (5)	0.0882 (9)	0.1105 (9)	0.0064 (5)	0.0323 (5)	−0.0146 (7)
O2	0.0473 (5)	0.0544 (7)	0.1169 (8)	0.0028 (4)	0.0349 (5)	−0.0095 (6)
O3	0.0502 (5)	0.0530 (6)	0.1159 (8)	−0.0031 (5)	0.0324 (5)	−0.0166 (6)
C7	0.0549 (7)	0.0635 (10)	0.0597 (7)	0.0154 (7)	0.0216 (6)	0.0049 (7)
C8	0.0784 (10)	0.0775 (13)	0.0775 (10)	0.0292 (10)	0.0297 (8)	−0.0028 (9)
C9	0.1128 (15)	0.0655 (12)	0.0739 (10)	0.0248 (11)	0.0296 (10)	−0.0121 (9)
C10	0.0967 (13)	0.0645 (12)	0.0772 (11)	−0.0022 (10)	0.0198 (9)	−0.0197 (9)
C11	0.0634 (8)	0.0604 (10)	0.0672 (9)	0.0032 (7)	0.0177 (7)	−0.0063 (7)
C12	0.0505 (6)	0.0480 (8)	0.0521 (6)	0.0086 (6)	0.0176 (5)	0.0059 (6)
C13	0.0471 (6)	0.0451 (8)	0.0719 (8)	0.0040 (6)	0.0252 (6)	0.0047 (7)

### Geometric parameters (Å, °)

**Table:**

N1—C1	1.3510 (15)	C6—H6C	0.94 (3)
N1—C5	1.3638 (17)	O1—C7	1.3567 (19)
N1—H1N1	1.030 (18)	O1—H1O1	1.03 (2)
N2—C1	1.3279 (18)	O2—C13	1.2467 (15)
N2—H1N2	0.884 (18)	O3—C13	1.2710 (16)
N2—H2N2	0.97 (2)	C7—C8	1.393 (2)
C1—C2	1.405 (2)	C7—C12	1.4006 (18)
C2—C3	1.359 (2)	C8—C9	1.365 (3)
C2—H2A	0.950 (15)	C8—H8A	0.938 (19)
C3—C4	1.413 (2)	C9—C10	1.377 (3)
C3—H3A	1.011 (18)	C9—H9A	0.99 (2)
C4—C5	1.352 (2)	C10—C11	1.384 (2)
C4—C6	1.500 (2)	C10—H10A	0.94 (2)
C5—H5A	0.960 (13)	C11—C12	1.391 (2)
C6—H6A	0.99 (2)	C11—H11A	0.962 (15)
C6—H6B	1.01 (3)	C12—C13	1.494 (2)
			
C1—N1—C5	122.26 (13)	H6A—C6—H6C	113.4 (17)
C1—N1—H1N1	118.8 (8)	H6B—C6—H6C	108 (2)
C5—N1—H1N1	118.9 (8)	C7—O1—H1O1	101.7 (11)
C1—N2—H1N2	117.6 (11)	O1—C7—C8	118.34 (13)
C1—N2—H2N2	118.2 (10)	O1—C7—C12	122.04 (13)
H1N2—N2—H2N2	124.1 (15)	C8—C7—C12	119.62 (15)
N2—C1—N1	118.50 (13)	C9—C8—C7	120.52 (16)
N2—C1—C2	123.91 (12)	C9—C8—H8A	124.5 (13)
N1—C1—C2	117.58 (11)	C7—C8—H8A	114.8 (13)
C3—C2—C1	119.91 (12)	C8—C9—C10	121.02 (18)
C3—C2—H2A	122.5 (9)	C8—C9—H9A	119.2 (11)
C1—C2—H2A	117.6 (9)	C10—C9—H9A	119.8 (11)
C2—C3—C4	121.70 (14)	C9—C10—C11	118.87 (18)
C2—C3—H3A	121.2 (9)	C9—C10—H10A	122.2 (13)
C4—C3—H3A	117.1 (9)	C11—C10—H10A	118.9 (13)
C5—C4—C3	116.52 (12)	C10—C11—C12	121.65 (15)
C5—C4—C6	121.61 (14)	C10—C11—H11A	120.0 (10)
C3—C4—C6	121.86 (16)	C12—C11—H11A	118.3 (9)
C4—C5—N1	122.02 (12)	C11—C12—C7	118.32 (13)
C4—C5—H5A	126.5 (8)	C11—C12—C13	120.92 (12)
N1—C5—H5A	111.4 (8)	C7—C12—C13	120.75 (12)
C4—C6—H6A	111.8 (13)	O2—C13—O3	123.17 (13)
C4—C6—H6B	108.9 (13)	O2—C13—C12	119.90 (12)
H6A—C6—H6B	102.8 (18)	O3—C13—C12	116.93 (11)
C4—C6—H6C	111.5 (14)		
			
C5—N1—C1—N2	179.21 (12)	C8—C9—C10—C11	−0.5 (3)
C5—N1—C1—C2	−0.82 (18)	C9—C10—C11—C12	−0.3 (3)
N2—C1—C2—C3	−178.87 (13)	C10—C11—C12—C7	1.0 (2)
N1—C1—C2—C3	1.16 (19)	C10—C11—C12—C13	−177.95 (14)
C1—C2—C3—C4	−0.7 (2)	O1—C7—C12—C11	179.29 (13)
C2—C3—C4—C5	−0.1 (2)	C8—C7—C12—C11	−0.8 (2)
C2—C3—C4—C6	178.54 (15)	O1—C7—C12—C13	−1.8 (2)
C3—C4—C5—N1	0.46 (19)	C8—C7—C12—C13	178.09 (13)
C6—C4—C5—N1	−178.17 (14)	C11—C12—C13—O2	−1.0 (2)
C1—N1—C5—C4	0.00 (19)	C7—C12—C13—O2	−179.86 (12)
O1—C7—C8—C9	179.92 (15)	C11—C12—C13—O3	178.39 (13)
C12—C7—C8—C9	0.0 (2)	C7—C12—C13—O3	−0.49 (19)
C7—C8—C9—C10	0.7 (3)		

### Hydrogen-bond geometry (Å, °)

**Table:**

*D*—H···*A*	*D*—H	H···*A*	*D*···*A*	*D*—H···*A*
N1—H1N1···O3	1.03 (2)	1.65 (2)	2.678 (2)	174.6 (13)
N2—H1N2···O2^i^	0.884 (18)	1.987 (17)	2.852 (2)	165.4 (14)
N2—H2N2···O2	0.97 (2)	1.90 (2)	2.872 (2)	179 (2)
O1—H1O1···O3	1.03 (2)	1.55 (2)	2.515 (2)	155 (2)
C5—H5A···O1^ii^	0.961 (14)	2.598 (14)	3.518 (3)	160.2 (10)

Symmetry codes: (i) −*x*, *y*+1/2, −*z*+1/2; (ii) −*x*+1, *y*+1/2, −*z*+1/2.
